# Supplementary material for: Analyzing online public commentary responding to the announcement of deemed consent organ donation legislation in the Canadian province of Nova Scotia
Source: PLoS One. 2022 Dec 15;17(12):e0278983. doi: 10.1371/journal.pone.0278983 (PMC9754165; doi:10.1371/journal.pone.0278983)
Supplement: S1 Text — Negative comment coding categories. (DOCX) [file pone.0278983.s001.docx]

**Negative comment coding categories**

Based on a complete initial reading of all negative comments, a categorization of themes and arguments was constructed. Each of these categories was non-mutually exclusive for each negative comment. Either the following category was present or non-present in each of the comments. The categories and their respective descriptions are as follows:

1. Government power (government usurping the power of the citizens; taking away citizens’ choice, exerting power over citizens, etc.)
2. Harvesting (using the word “harvest” or similar derivatives such as “chop up”, “take what they want”)
3. Ownership (a citizen loses ownership of their body (corpse), the government takes ownership of the body (corpse) -*included in this category was the idea that government ownership may supersede the ownership of family members)
4. Profits (financial gains were going to be made as a result of presumed consent)
5. Legal (issues around legality including the mention of the Constitution, the Charter of Rights, and the courts (i.e. the Supreme court, lawsuits))
6. Doctors’ treatment of patients (Doctors would not treat patients as well as others because they would be eager to use organs)
7. Pro donation (those stating that they were in favour of donation (i.e. had signed up to be a donor in the past) but disagreed with presumed consent (in some cases may not be willing to be a donor in the future (would opt-out))
8. Comparisons (making analogies between other situations where consent was required and/or presumed (i.e. negative billing; cable and internet companies, etc.)
9. Dystopia (pop culture dystopian references)
10. Infrastructure (concerns that the health care system could not handle an increase in donations and/or that the services are generally ill-equipped and poorly-managed)
11. Liberals (mentioning of “Liberals”, “Trudeau” or similar derivatives)
12. Other countries (mentioning other countries like China or Russia in disparaging ways including political regimes such as communism or fascism)
13. Procedures (concerns around how the presumed consent processes (and donation because of presumed consent) would take place (i.e. for those visiting the province, how the population would be notified of opting-out, time limits set on opting-out, concerns around determining death, etc.)
14. Religions/Cultures (concerns for members of religions or for cultural groups who may not be aware of the issues or who may not possess strong English language skills)
15. Consultation (lack of consultation with the public, “first I have heard of this”, etc.)
16. Maintain Opt-in (arguments that Opt-in system is the right way to manage donations and/or current opt-in system needs to be improved)
17. Morally wrong (mention of morals or ethics)
18. General (without fitting into other categories “wrong”, “don’t like this”, etc.)
