# Supplementary material for: Analyzing online public commentary responding to the announcement of deemed consent organ donation legislation in the Canadian province of Nova Scotia
Source: PLoS One. 2022 Dec 15;17(12):e0278983. doi: 10.1371/journal.pone.0278983 (PMC9754165; doi:10.1371/journal.pone.0278983)
Supplement: S1 Table — The three most liked, disliked, and replied-to comments from each article. (DOCX) [file pone.0278983.s002.docx]

**The three most liked, disliked, and replied-to comments from each article**

| Comment type | Article 1 (# of likes, dislikes, or replies) | Article 2 (# of likes, dislikes, or replies) |
| --- | --- | --- |
| Most liked | “I think as long as your organs are in good conditions they should be used to save lives, no use taking them with you....” (63) | “Simply opt out. Even if you fail to opt out, family still must confirm your intention so there is simply no reason for opposition.(31) |
| 2^nd^ most liked | “Good - those who don't want their organs donated can opt out.” (52) | “Why is it that some people line up and shout to the rooftops to save the life of a bunch of cells in the womb, but when it comes to saving a life with the donation of their organs...hands-off man...” (27) |
| 3^rd^ most liked | “finally a smart decision from a North American government” (48) | “"On CBC News' Facebook, Vicki Rewakoski shared a similar sentiment. "Although I am an organ donor, I don't believe that this is right! It takes away a person's right to choose."" Is basic understanding now gone the same way as common sense? You *STILL* have the right to choose... but if you don't make a decision, then you're a donor, just as it should be. Get over yourself.” (26) |
| Most disliked | “Have always opted to be a donor & now I'll be opting out entirely. Government should stay out of my choices.” (32) | “It has often been said that, 'The only two guarantees in life are death and taxes'. We need a fraction of the government's involvement that currently exists in our lives, and only one political party understands this. Vote Conservative.” (20) |
| 2^nd^ most disliked | “Never confuse the word politician with smart. Not only is this a recipe for a lawsuit but blatantly unconstitutional. Its about time the federal government passes a law that makes it illegal for politicians to knowingly pass laws which are not legal. A mandatory minimal jail sentence for this kinf of breach seem wholly appropriate.”(29) | “Presumed consent is wrong ... If I wanted to donate I would, but I am sorry I do not feel cofortable with the idea.[sic]” (17) |
| 3^rd^ most disliked | “What right does the government/state have to my body? I'm not against the practice of organ donation, but this is a step too far, it's an infringement on personal freedom. I'm curious if this is at all contradictory to the Charter of Rights and Freedoms, I know it certainly goes against many religious and cultural beliefs/practices.” (26) | “I don't agree with it. It makes us property of the state, and that is a slippery slope. Technically. Slaves.” (16). |
| Most replies | *Same as 3^rd^ most disliked comment (25)* | “If YOU do not wish to donate YOUR organs, then opt out. No one is forcing you to donate your organs against your wishes.” (22) |
| 2^nd^ most replies | “You. Don't. Have. To. Donate. OPT OUT & shuddup - NO ONE will suffer from this, A LOT of people will be able to LIVE. Cheers to the people that are so vehemently against this, you would rather have people suffer. IT'S A CHOICE.” (24) | “This is disgusting. I'm opting out, not because of agreeing or disagreeing with organ donation but because the government has no place assuming anybody's consent, and it's bad lawmaking all around. And just because you can "opt out" doesn't change that people are being guilt-tripped and coerced into "giving" that way - whereas before it was assumed and thus kept private, now we have to formally seek to opt out from the government, which is socially viewed as selfish. What's more selfish are some of the comments I'm seeing from people who are greedy to get "spare parts, because dead is dead". Maybe people would be more likely to donate organs if people stopped talking about them as if they are pieces of meat at the grocery store to be reaped and harvested at any chance we get? It's selfish to be so desperate for transplants that you're willing to presume consent from who might be an unwilling donor, and to see them as "parts" rather than individual human beings, much for selfish than it is to opt out of donation.” (17) |
| 3^rd^ most replies | *Same as most disliked comment* (23) | “I thought the government spent all this time and energy to tell us that implied consent doesn't exist and you need explicit consent. another example of do as i say not as i do.” (14) |
