# Supplementary material for: Analyzing online public commentary responding to the announcement of deemed consent organ donation legislation in the Canadian province of Nova Scotia
Source: PLoS One. 2022 Dec 15;17(12):e0278983. doi: 10.1371/journal.pone.0278983 (PMC9754165; doi:10.1371/journal.pone.0278983)
Supplement: S2 Table — Summary of participants’ comments based on negative, positive and neutral comment contribution. (DOCX) [file pone.0278983.s003.docx]

**Summary of participants’ comments based on negative, positive and neutral comment contribution**

| Participants | Total (with duplicates removed) |
| --- | --- |
| Number of unique participants contributing… |  |
| negative comments | 184 (17 in both) |
| positive comments | 145 (9 in both) |
| neutral comments | 95 (1 in both) |
| Total | 425 |
| Average number of comments per participant for… |  |
| negative comments | 1.84 |
| positive comments | 1.18 |
| neutral comments | 1.24 |
|  |  |
| Number of comments by participants contributing 1 (*N*=301)… |  |
| negative comment | 132 (43.6%) |
| positive comment | 116 (38.5%) |
| neutral comment | 53 (17.6%) |
|  |  |
| Number comments by participants contributing 2 (*N*=102)… |  |
| negative comments | 45 (44.1%) |
| positive comments | 32 (31.4%) |
| neutral comments | 25 (24.5%) |
|  |  |
| Number of comments by participants contributing more than 2 (*N*=226)… |  |
| negative comments | 162 (71.7%) |
| positive comments | 24 (10.6%) |
| neutral comments | 40 (17.7%) |
|  |  |
| Number of comments by the five most active participants for… |  |
| negative comments | 93 (27.3%) |
| positive comments | 18 (10.4%) |
| neutral comments | 16 (13.5%) |
|  |  |
| Number of most active participants to account for 27% of total comments for… |  |
| negative comments | 5 (2.7%) |
| positive comments | 20 (13.7%) |
| neutral comments | 13 (13.7%) |
|  |  |
| Total comments and replies for five most active participants contributing at least 2… |  |
| negative comments | 283 (10.6%) |
| positive comments | 115 (4.3%) |
| neutral comments | 154 (5.8%) |
