# Supplementary material for: Analyzing online public commentary responding to the announcement of deemed consent organ donation legislation in the Canadian province of Nova Scotia
Source: PLoS One. 2022 Dec 15;17(12):e0278983. doi: 10.1371/journal.pone.0278983 (PMC9754165; doi:10.1371/journal.pone.0278983)
Supplement: S3 Table — Text examples for each negative comment category type. (DOCX) [file pone.0278983.s004.docx]

**Text examples for each negative comment category type**

| **Coding Category** “examples” |
| --- |
| **Gov. usurp power**   - **“**Any time government takes away YOUR choice and makes it for you BEWARE.” - “the government simply will not stop taking from the people...…..” - “How much State intrusion into personal freedom is acceptable?” |
| **Ownership**   - “I am all for organ donation but I don't agree with the government basically 'owning' our organs after death unless we have opted out.” - “This does nothing more than reinforce the notion that the government believes they literally own you.” |
| **Legal**   - “Not only is this a recipe for a lawsuit but blatantly unconstitutional. Its about time the federal government passes a law that makes it illegal for politicians to knowingly pass laws which are not legal. A mandatory minimal jail sentence for this kinf of breach seem wholly appropriate. [sic]” - “There’s a reason this doesn’t exist. It won’t pass a charter case to the Supreme Court.” |
| **"Harvest"**   - “So they are going to start harvesting us for parts.” - “Organ donation is a good thing, I know people who have received the gift of life and health....harvesting is another matter.” |
| **Procedures**   - **“**I'd like to know exactly how the procedure in the hospital works as it conjures up a picture of people keeping the dying on life support just long enough to have their organs harvested, or organ vultures waiting at bedsides or worse” - “This better not turn into a annual or every five year "opt out" program, where if you forget to opt out and die, then too bad for your family "you're getting chopped up"…” |
| **General**   - “I'm not from NS, and it does not affect me (directly), and I oppose this, no matter what anyone says” - “Organ donation is good and people should do it. But the decision should be a vluntary decision, not presumed consent.[sic]” |
| **Liberals**   - **“**Everything these liberals touch while we are alive gets messed up,now they are doing it when we are dead.” - “NS liberal motto on this invasion…. ‘dear de-parted’. They can’t find or afford enough doctors to cover provincial health needs.” |
| **Profits**   - “the body parts will be sold outside of the province and the country, this is purely a money making venture.. this will be one of the largest scandals in Nova Scotia history.. I can't wait to get the goods on this...” - “you organs will now go all over the world... big money!” |
| **Pro-donation**   - “Have always opted to be a donor & now I'll be opting out entirely. Government should stay out of my choices.” - “Organ donation is a good thing, I know people who have received the gift of life and health....harvesting is another matter.” |
| **Comparisons (of consent)**   - “Not a fan of opt out programs. It's like volunteering by default. Blood banks across the nation are always struggling for donations. Heck, lets get everybodys blood groupand line them up.[sic]” - “I find the entire idea of negative optioning rather repulsive. What ever happened to my body my choice?” - When companies employ a negative billing policy (you specifically have to opt out/deny or you will be included/charged), we clamour for the gov't to put an end to it. But when a gov't employs a negative billing policy to chop up our corpses, s'alriiiiight...” |
| **Doctors**   - “as I lay on an operating table and a doctor gives me a less than 50% chance of survival, they will choose to :assist me" in passing on, so they can harvest my organs at their "peak ripeness" and ensure they can garner the highest price for their sale.” - “This law gives too much decision making to the doctors! Based on decisions I have seen surgeons make over the past 2 years, I just do not trust them!” |
| **Improve current**   - “I think the government should instead opt to have programs to sign more people up. Have it on census forms, all pages of all government web sites, in the footer message of all government emails and mail. On tax forms, electronic and otherwise, build a database.” - “…why not go back to having a donor card. If I wanted to donate I would then fill out a card.” |
| **Other countries**   - **“**First the gov't intrudes into your mind telling you what to think with media control: then the gov't intrudes into your body WITH OUT YOUR CONSENT....more like Russia/China/DYNK every day here in Canada!!!” - “NS isn’t England or Spain or any other country… this about the will and wishes of the individual.” |
| **Morally wrong**   - **“**This is just plain wrong. The ends however worthy never justify unethical and morally repugnant means.” - “Presumed consent is morally wrong, and subject to zillions of bureaucratic bungles e.g. countless of laptops with personal health data for millions of Canadians left in bars or on park benches.” |
| **Religions/ Cultures**   - “I wonder how they intend to get around religious and cultural beliefs with this.” - “I know it certainly goes against many religious and cultural beliefs/practices.” |
| **Consultation**   - **“**Btw, citizens of the province found out about this in the news. How is that for democracy.” - “This government has not been proactive in discussions with the public.. nothing about it until the news article. They’ve not had the health community bring it up during doctor visits, when visiting the hospital for tests, not a letter or a call. It isn’t like Nova Scotians are unable to be reached. It is however that the government can’t afford to inform the public, we are living in a poor house.” |
| **Infrastructure**   - **“**Oh how nice. A province with one of the highest tax rates, unemployment, a shortage of doctors, and closed emergency wards wants to put itself in the history books! Work on your Medicare system and looking after your citizens instead of gouging them first, idiots!” - “We have 3 transplant surgeons in all of Atlantic Canada. There is zero capacity for the large increase in transplants so where are the organs going to be used.” |
| **Dystopia**   - **“**Next up, Soylent Green.” - “"Soylent green is people" - “should not be implied consent too much big brother” |
