# Supplementary material for: Analyzing online public commentary responding to the announcement of deemed consent organ donation legislation in the Canadian province of Nova Scotia
Source: PLoS One. 2022 Dec 15;17(12):e0278983. doi: 10.1371/journal.pone.0278983 (PMC9754165; doi:10.1371/journal.pone.0278983)
Supplement: Attachment — Submitted filename: Response to reviewers.docx [file pone.0278983.s005.docx]

Dear Ronaldo Menezes, Academic Director,

Thank you for the peer review of our manuscript. The few changes asked of us by peer reviewers are valuable considerations, and making the requested changes has strengthened the manuscript. Please see a point-by-point response to the issues raised by both reviewers. The accompanying manuscript includes the new modifications.

Sincerely,

Alessandro Marcon (on behalf of all authors)

**Response to reviewer comments**

**Reviewer 1 comment: (1) It seems not to be the case that Brazil implemented presumed consent legislation. Please, double check the sentence “Some countries, such as Singapore, Brazil, and Chile11,12,13 implemented presumed consent legislation but were unsuccessful at increasing donation and transplantation rates”**

Response: We’re a bit unsure of this critique. In this statement we wanted to state that in the past the countries listed implemented a version of deemed or presumed consent and were unsuccessful at raising rates. To our knowledge and as evidenced in our cited examples as well as other research, Brazil most certainly did implement presumed consent and faced significant backlash from the public after doing so. (For more examples please see: <https://www.thelancet.com/journals/lancet/article/PIIS0140-6736(05)60767-2/fulltext> ; or <https://link.springer.com/article/10.1007/s10620-019-05483-z> or <https://www.ncbi.nlm.nih.gov/pmc/articles/PMC3363073/>). We don’t think this is an opinion or a controversial interpretation, rather common knowledge. Please advise if our statement is incorrectly phrased.

**Reviewer 1 comment: (2) What is the data collection period? The dates of the two articles published on CBC (April 2nd and April 4th, 2019) are clearly stated but what is the period regarding all the 2663 respective comments and replies?**

Response: We collected the data on May 24^th^ 2019. We have included this statement in the methods.

**Reviewer 1 comment: (3) Is the code of program that collected the comments and replies available? I would suggest the authors to provide more detail about this program and its workflow.**

Response: We are providing the git hub link to the program, which has been available to the public since it was created. We think our description adequately describes what the program does as it as a simple scraper. If anyone wishes to have further coding details, the link can be accessed.

Complete script available here: <https://giters.com/philbot9/python-cbc-comment-scraper/watchers>

We have also added the following line for additional clarity: “The complete data sets were organized in CSV files.”

**Reviewer 1 comment: (4) I wound whether bots (fake accounts) played a role in “the negative commentary was greatly influenced by small group of dedicated commentators”.**

Response: This was also something our team discussed when conducting the analysis, but conducting analysis on participant accounts fell outside of the purview of this project. We therefore did not try running one of the few bot detection programs that some researchers have developed. As mentioned earlier, to acknowledge the importance of this point, we have included the following line in our discussion:

“It is possible that automated accounts, or “bots,” were contributing to the negativity in the discussions, but analyzing user accounts fell outside of the purview of this research. Future research on the topic could make use of developing software to estimate bot activity in such discussions. Future research could also examine public discussions on other social media sites such as Facebook, Reddit, or Twitter to provide additional insights into the discussions taking place.”

**Reviewer 2 comment (1): It would be great, in the introduction, to show the rates, in number, of organ donation from Nova Scotia and other Canadian provinces. Also, the name of the software that you use to collect the data.**

Response: We do not feel that including those specific numbers would add consider value and that including them would clutter the introduction. Those wishing to see the numbers can easily access the data in the reference we have provided, which links to an open-access database. If the editors of the journal believe it is good idea to include these numbers, we would be happy to include them.

We have provided a link to the software we developed to download comments from the CBC’s website.

**Reviewer 2 comment (2): I did not fully understand the definition of positive, negative, and neutral comments in the methods section**.

Response: We agree with this comment and have added the following to the Methods section, which should provide additional clarity:

“Comments supporting and promoting the new legislation as well as donation and transplantation broadly were deemed positive whereas commenting critiquing the new legislation as well as donation and transplantation broadly were deemed negative. Comments which did not exhibit negativity or positivity, or which did not present a clearly defined negative or positive position, were coded as neutral.”

**Reviewer 2 Comment (3): Why did you collect the data in three days (April 2nd and April 4th, 2019)? Do you think that it might be a bias toward negative comments?**

Response: We appreciate this inquiry, but we do not think our data collection procedures would indicate any bias towards negative comments. We provided ample time for discussion to take place. If anything, more negativity might have been removed from the discussions as moderators would likely have blocked and removed exceedingly aggressive, offensive, or hateful comments based on their criteria for doing so.
